# *catena*-Poly[(di­aqua­cadmium)-μ-iminodi­acetato-κ^4^ *O*,*N*,*O*′:*O*′′]

**DOI:** 10.1107/S1600536813017121

**Published:** 2013-06-26

**Authors:** Gang-Hong Pan, Jin-Niu Tang, Zhong-Jing Huang, Yi-Fan Liao, Bo-Fa Mo

**Affiliations:** a College of Chemistry and Chemical Engineering, Guangxi University for Nationalities, Nanning 530006, People’s Republic of China

## Abstract

In the title compound, [Cd(C_4_H_5_NO_4_)(H_2_O)_2_]_*n*_, the Cd^II^ atom exhibits a distorted octa­hedral coordination geometry, defined by one N atom and three O atoms from two iminodi­acetate (IDA) ligands and two water molecules. The tridentate IDA ligand additionally bridges *via* one of its carboxylate O atoms to another Cd^II^ atom, thus forming a zigzag chain along [001]. A three-dimensional network is completed by inter­molecular O—H⋯O and N—H⋯O hydrogen bonds.

## Related literature

For background to Cd^II^ complexes, see: Brusau *et al.* (2001 ▶). For related structures, see: Su & Xu (2005 ▶); Zhang & Lu (2004 ▶).
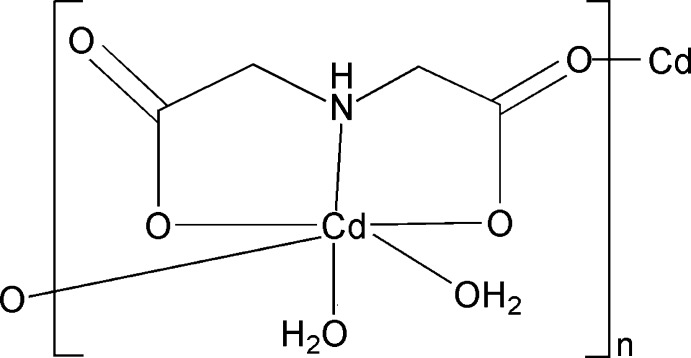


## Experimental

### 

#### Crystal data

[Cd(C_4_H_5_NO_4_)(H_2_O)_2_]
*M* *_r_* = 279.52
Orthorhombic, 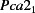

*a* = 14.6600 (3) Å
*b* = 5.4905 (2) Å
*c* = 9.7928 (3) Å
*V* = 788.23 (4) Å^3^
*Z* = 4
Mo *K*α radiation
μ = 2.76 mm^−1^
*T* = 296 K
0.22 × 0.17 × 0.16 mm

#### Data collection

Bruker SMART 1000 CCD diffractometer
Absorption correction: multi-scan (*SADABS*; Sheldrick, 1996 ▶) *T*_min_ = 0.582, *T*_max_ = 0.666
3775 measured reflections
1303 independent reflections
1173 reflections with *I* > 2σ(*I*)
*R*_int_ = 0.036

#### Refinement

*R*[*F* ^2^ > 2σ(*F* ^2^)] = 0.047
*wR*(*F* ^2^) = 0.164
*S* = 1.09
1303 reflections
109 parameters
1 restraint
H-atom parameters constrained
Δρ_max_ = 2.05 e Å^−3^
Δρ_min_ = −1.23 e Å^−3^
Absolute structure: Flack (1983 ▶), 566 Friedel pairs
Flack parameter: 0.04 (14)

### 

Data collection: *SMART* (Bruker, 2007 ▶); cell refinement: *SAINT* (Bruker, 2007 ▶); data reduction: *SAINT*; program(s) used to solve structure: *SHELXS97* (Sheldrick, 2008 ▶); program(s) used to refine structure: *SHELXL97* (Sheldrick, 2008 ▶); molecular graphics: *DIAMOND* (Brandenburg, 1999 ▶); software used to prepare material for publication: *SHELXTL* (Sheldrick, 2008 ▶).

## Supplementary Material

Crystal structure: contains datablock(s) I, global. DOI: 10.1107/S1600536813017121/hy2628sup1.cif

Structure factors: contains datablock(s) I. DOI: 10.1107/S1600536813017121/hy2628Isup2.hkl

Additional supplementary materials:  crystallographic information; 3D view; checkCIF report

## Figures and Tables

**Table 1:** Hydrogen-bond geometry (Å, °)

*D*—H⋯*A*	*D*—H	H⋯*A*	*D*⋯*A*	*D*—H⋯*A*
O5—H5*A*⋯O1^i^	0.85	1.84	2.685 (15)	176
O5—H5*B*⋯O6^ii^	0.85	2.03	2.880 (15)	176
O6—H6*A*⋯O2^iii^	0.85	1.81	2.649 (14)	168
O6—H6*B*⋯O3^ii^	0.85	1.91	2.750 (15)	168
N1—H1⋯O2^i^	0.91	2.05	2.953 (16)	174

Symmetry codes: (i) 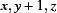; (ii) 
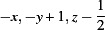
; (iii) 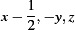.

**Fig. 1.**
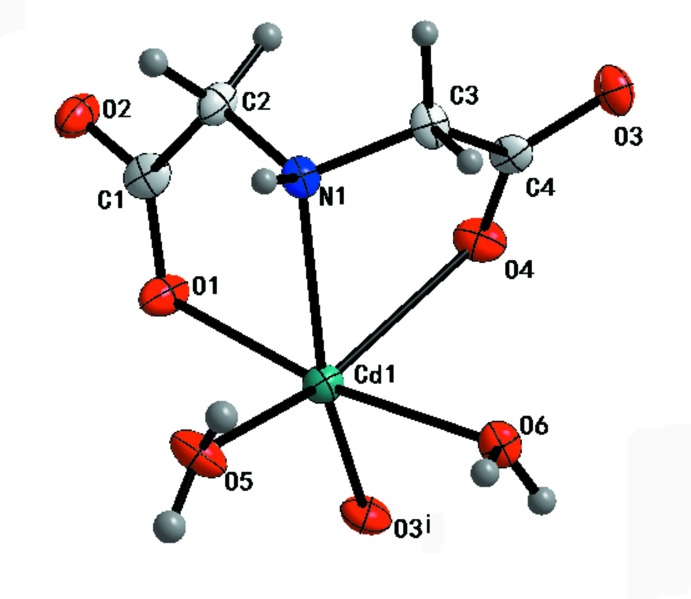
The asymmetric unit of the title compound. Displacement ellipsoids are drawn at the 30% probability level. [Symmetry code: (i) -x, -y, -1/2+z.]

**Fig. 2.**
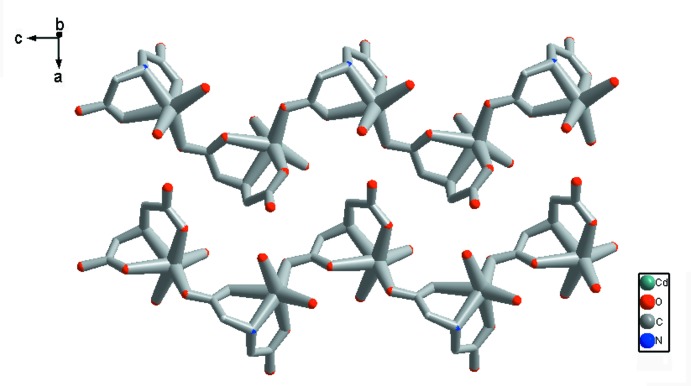
Perspective view of the chains in the title compound.

**Fig. 3.**
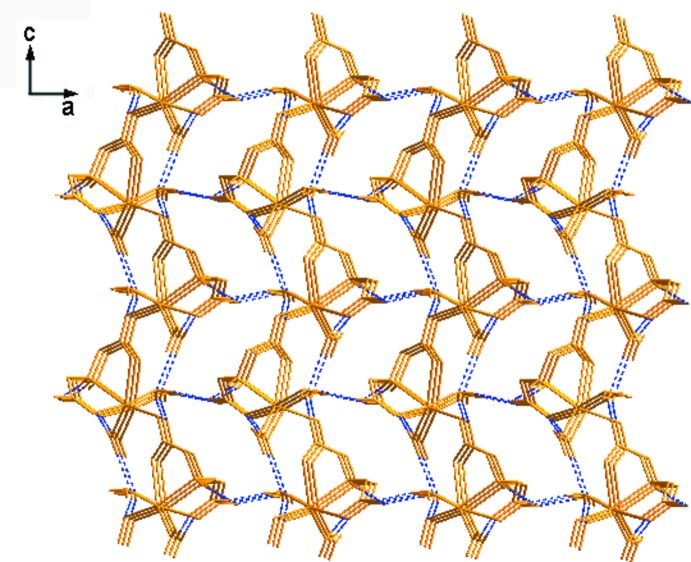
Crystal packing of the title compound, showing intermolecular hydrogen-bonding interactions (dashed lines).

## supplementary crystallographic information

### Comment

Cd(II) ion with d^10^ electronic configuration exhibits a wide variety of coordination geometries and modes, which can induce versatile structural topologies (Brusau *et al.*, 2001). A large number of metal-organic compounds based on Cd(II) have been reported. However, to the best of our knowledge, only the structure of a Cd(II) complex with benzimidazole and iminodiacetate (IDA) ligands has been reported so far (Su & Xu, 2005). We report here the structure of a Cd(II) iminodiacetate coordination polymer.

In the title complex (Fig. 1), the Cd^II^ atom exhibits a distorted octahedral coordination geometry defined by one N atom and two atoms from an IDA ligand, one O atom from another IDA and two water molecules. The five membered chelating ring generated by O1—C1—C2—N1—Cd1 is nearly planar, with a largest deviation of -0.093 (16) from C2 to the mean plane, while the O4—C4—C3—N1—Cd1 chelating ring shows largest deviations of -0.440 (16) for C3 and 0.304 (11) for N1 in the opposite directions from the mean plane. The dihedral angle between the two chelating ring planes is 82.4 (3)°. The bond distances of Cd—O and Cd—N are comparable to those in [(benzimidazole)_3_(IDA)Cd.2H_2_O] (Su & Xu, 2005). However, these bond distances are 0.06–0.19Å longer than the values in a reported Mn(II) analogs (Zhang & Lu, 2004). The IDA ligand bridges two Cd^II^ atoms, forming a zigzag chain along [001] (Fig. 2). A three-dimensional network is completed by intermolecular O—H···O and N—H···O hydrogen bonds (Fig. 3).

### Experimental

A mixture of iminodiacetic acid (0.067 g, 0.5 mmol), CdSO_4_.8H_2_O (0.208 g, 1 mmol), NaOH (0.040 g, 1 mmol) and water (15 ml) was sealed in a Teflon-lined stainless steel vessel (25 cm^3^), and then the vessel was heated at 403 K for 3 days. After the mixture was slowly cooled to room temperature, colorless block-shaped crystals of the title compound were obtained. Analysis, calculated for C_4_H_9_CdNO_6_: C 17.19, H 3.25, N 5.01%; found: C 17.16, H 3.33, N 5.08%.

### Refinement

H atoms bonded to C and N atoms were positioned geometrically and refined as riding atoms, with C—H = 0.97 and N—H = 0.91 Å and with *U*_iso_(H) = 1.2*U*_eq_(C, N). H atoms of water molecules were located in a difference Fourier map and refined as riding, with O—H = 0.85 Å and *U*_iso_(H) = 1.2*U*_eq_(O). The maximum remaining electron density is found 1.13 Å from Cd1 and the minimum density 1.43 Å from O2.

### Crystal data

**Table:**

[Cd(C_4_H_5_NO_4_)(H_2_O)_2_]	*F*(000) = 544
*M**_r_* = 279.52	*D*_x_ = 2.355 Mg m^−^^3^
Orthorhombic, *P**c**a*2_1_	Mo *K*α radiation, λ = 0.71073 Å
Hall symbol: P 2c -2ac	Cell parameters from 1202 reflections
*a* = 14.6600 (3) Å	θ = 2.8–22.1°
*b* = 5.4905 (2) Å	µ = 2.76 mm^−^^1^
*c* = 9.7928 (3) Å	*T* = 296 K
*V* = 788.23 (4) Å^3^	Block, colorless
*Z* = 4	0.22 × 0.17 × 0.16 mm

### Data collection

**Table:**

Bruker SMART 1000 CCD diffractometer	1303 independent reflections
Radiation source: fine-focus sealed tube	1173 reflections with *I* > 2σ(*I*)
Graphite monochromator	*R*_int_ = 0.036
φ and ω scans	θ_max_ = 25.0°, θ_min_ = 2.8°
Absorption correction: multi-scan (*SADABS*; Sheldrick, 1996)	*h* = −17→16
*T*_min_ = 0.582, *T*_max_ = 0.666	*k* = −6→6
3775 measured reflections	*l* = −9→11

### Refinement

**Table:**

Refinement on *F*^2^	Secondary atom site location: difference Fourier map
Least-squares matrix: full	Hydrogen site location: inferred from neighbouring sites
*R*[*F*^2^ > 2σ(*F*^2^)] = 0.047	H-atom parameters constrained
*wR*(*F*^2^) = 0.164	*w* = 1/[σ^2^(*F*_o_^2^) + (0.0941*P*)^2^ + 9.9695*P*] where *P* = (*F*_o_^2^ + 2*F*_c_^2^)/3
*S* = 1.09	(Δ/σ)_max_ < 0.001
1303 reflections	Δρ_max_ = 2.05 e Å^−^^3^
109 parameters	Δρ_min_ = −1.23 e Å^−^^3^
1 restraint	Absolute structure: Flack (1983), 566 Friedel pairs
Primary atom site location: structure-invariant direct methods	Flack parameter: 0.04 (14)

### Special details

**Table:**

Geometry. All esds (except the esd in the dihedral angle between two l.s. planes) are estimated using the full covariance matrix. The cell esds are taken into account individually in the estimation of esds in distances, angles and torsion angles; correlations between esds in cell parameters are only used when they are defined by crystal symmetry. An approximate (isotropic) treatment of cell esds is used for estimating esds involving l.s. planes.
Refinement. Refinement of F^2^ against ALL reflections. The weighted R-factor wR and goodness of fit S are based on F^2^, conventional R-factors R are based on F, with F set to zero for negative F^2^. The threshold expression of F^2^ > 2sigma(F^2^) is used only for calculating R-factors(gt) etc. and is not relevant to the choice of reflections for refinement. R-factors based on F^2^ are statistically about twice as large as those based on F, and R- factors based on ALL data will be even larger.

### Fractional atomic coordinates and isotropic or equivalent isotropic displacement parameters (Å^2^)

**Table:**

	*x*	*y*	*z*	*U*_iso_*/*U*_eq_	
Cd1	0.05597 (5)	0.13193 (15)	0.74648 (19)	0.0292 (3)	
O1	0.1596 (8)	−0.1730 (19)	0.7032 (11)	0.040 (3)	
O2	0.2833 (7)	−0.3435 (19)	0.7786 (12)	0.043 (4)	
O3	0.0546 (7)	0.1227 (18)	1.1908 (13)	0.037 (3)	
O4	0.0357 (7)	−0.022 (2)	0.9811 (12)	0.039 (2)	
O5	0.1075 (9)	0.406 (2)	0.5878 (11)	0.042 (3)	
H5A	0.1248	0.5351	0.6279	0.051*	
H5B	0.0880	0.4441	0.5090	0.051*	
O6	−0.0422 (7)	0.4382 (19)	0.8246 (11)	0.032 (2)	
H6A	−0.0969	0.3891	0.8160	0.039*	
H6B	−0.0378	0.5765	0.7861	0.039*	
N1	0.1820 (8)	0.229 (2)	0.8812 (13)	0.027 (2)	
H1	0.2099	0.3623	0.8448	0.032*	
C1	0.2288 (10)	−0.184 (3)	0.7810 (15)	0.033 (4)	
C2	0.2468 (10)	0.026 (3)	0.8802 (17)	0.033 (3)	
H2A	0.2493	−0.0406	0.9718	0.040*	
H2B	0.3067	0.0920	0.8600	0.040*	
C3	0.1468 (10)	0.294 (3)	1.0168 (15)	0.027 (3)	
H3A	0.1204	0.4559	1.0135	0.033*	
H3B	0.1967	0.2967	1.0818	0.033*	
C4	0.0744 (10)	0.112 (2)	1.0644 (17)	0.027 (3)	

### Atomic displacement parameters (Å^2^)

**Table:**

	*U*^11^	*U*^22^	*U*^33^	*U*^12^	*U*^13^	*U*^23^
Cd1	0.0289 (5)	0.0255 (5)	0.0333 (5)	−0.0008 (3)	−0.0017 (7)	−0.0014 (6)
O1	0.035 (6)	0.032 (6)	0.054 (8)	0.005 (4)	−0.012 (5)	−0.017 (4)
O2	0.030 (5)	0.047 (6)	0.054 (11)	0.008 (4)	−0.004 (5)	−0.017 (5)
O3	0.046 (7)	0.031 (6)	0.033 (6)	−0.006 (4)	0.010 (4)	0.004 (4)
O4	0.046 (6)	0.029 (6)	0.041 (6)	−0.013 (5)	−0.007 (5)	0.001 (5)
O5	0.071 (9)	0.027 (6)	0.028 (6)	−0.012 (5)	−0.008 (5)	0.002 (4)
O6	0.043 (6)	0.020 (5)	0.034 (6)	0.000 (4)	0.003 (4)	0.001 (4)
N1	0.032 (6)	0.021 (5)	0.029 (6)	−0.003 (5)	0.002 (5)	−0.001 (5)
C1	0.033 (7)	0.021 (7)	0.044 (13)	−0.010 (6)	0.001 (6)	0.009 (6)
C2	0.029 (7)	0.027 (7)	0.043 (8)	0.003 (6)	−0.001 (6)	−0.011 (7)
C3	0.039 (8)	0.012 (6)	0.031 (8)	−0.005 (6)	0.006 (6)	−0.004 (5)
C4	0.031 (7)	0.016 (7)	0.032 (7)	0.000 (5)	−0.001 (6)	0.010 (6)

### Geometric parameters (Å, º)

**Table:**

Cd1—O3^i^	2.209 (10)	O5—H5B	0.8500
Cd1—O5	2.291 (11)	O6—H6A	0.8501
Cd1—O1	2.300 (11)	O6—H6B	0.8500
Cd1—N1	2.333 (12)	N1—C2	1.463 (18)
Cd1—O6	2.342 (10)	N1—C3	1.468 (18)
Cd1—O4	2.466 (12)	N1—H1	0.9100
O1—C1	1.270 (18)	C1—C2	1.53 (2)
O2—C1	1.186 (18)	C2—H2A	0.9700
O3—C4	1.27 (2)	C2—H2B	0.9700
O3—Cd1^ii^	2.209 (10)	C3—C4	1.531 (19)
O4—C4	1.24 (2)	C3—H3A	0.9700
O5—H5A	0.8500	C3—H3B	0.9700
			
O3^i^—Cd1—O5	119.4 (4)	C2—N1—C3	114.8 (12)
O3^i^—Cd1—O1	88.8 (4)	C2—N1—Cd1	109.6 (8)
O5—Cd1—O1	97.7 (4)	C3—N1—Cd1	106.8 (8)
O3^i^—Cd1—N1	150.0 (4)	C2—N1—H1	108.5
O5—Cd1—N1	88.4 (4)	C3—N1—H1	108.5
O1—Cd1—N1	75.4 (4)	Cd1—N1—H1	108.5
O3^i^—Cd1—O6	94.8 (4)	O2—C1—O1	124.1 (14)
O5—Cd1—O6	87.3 (4)	O2—C1—C2	117.0 (13)
O1—Cd1—O6	171.4 (4)	O1—C1—C2	118.8 (13)
N1—Cd1—O6	97.9 (4)	N1—C2—C1	117.8 (12)
O3^i^—Cd1—O4	85.7 (4)	N1—C2—H2A	107.9
O5—Cd1—O4	153.7 (4)	C1—C2—H2A	107.9
O1—Cd1—O4	90.1 (4)	N1—C2—H2B	107.9
N1—Cd1—O4	69.3 (4)	C1—C2—H2B	107.9
O6—Cd1—O4	82.4 (4)	H2A—C2—H2B	107.2
C1—O1—Cd1	116.9 (9)	N1—C3—C4	111.1 (12)
C4—O3—Cd1^ii^	112.2 (10)	N1—C3—H3A	109.4
C4—O4—Cd1	110.8 (9)	C4—C3—H3A	109.4
Cd1—O5—H5A	109.5	N1—C3—H3B	109.4
Cd1—O5—H5B	131.9	C4—C3—H3B	109.4
H5A—O5—H5B	108.3	H3A—C3—H3B	108.0
Cd1—O6—H6A	108.7	O4—C4—O3	124.4 (14)
Cd1—O6—H6B	116.8	O4—C4—C3	120.4 (14)
H6A—O6—H6B	108.1	O3—C4—C3	115.0 (13)

Symmetry codes: (i) −*x*, −*y*, *z*−1/2; (ii) −*x*, −*y*, *z*+1/2.

### Hydrogen-bond geometry (Å, º)

**Table:**

*D*—H···*A*	*D*—H	H···*A*	*D*···*A*	*D*—H···*A*
O5—H5*A*···O1^iii^	0.85	1.84	2.685 (15)	176
O5—H5*B*···O6^iv^	0.85	2.03	2.880 (15)	176
O6—H6*A*···O2^v^	0.85	1.81	2.649 (14)	168
O6—H6*B*···O3^iv^	0.85	1.91	2.750 (15)	168
N1—H1···O2^iii^	0.91	2.05	2.953 (16)	174

Symmetry codes: (iii) *x*, *y*+1, *z*; (iv) −*x*, −*y*+1, *z*−1/2; (v) *x*−1/2, −*y*, *z*.
